# Free-Standing CNT Film for Interlaminar Toughening: Insight into Infiltration and Thickness Effects

**DOI:** 10.3390/polym15173579

**Published:** 2023-08-29

**Authors:** Anran Fu, Yunfu Ou, Longqiang Wu, Yunxiao Zhang, Yiting Weng, Dongsheng Mao

**Affiliations:** 1 Key Laboratory of Marine Materials and Related Technologies, Zhejiang Key Laboratory of Marine Materials and Protective Technologies, Ningbo Institute of Materials Technology and Engineering, Chinese Academy of Sciences, Ningbo 315201, China; 2 School of Chemical Sciences, University of Chinese Academy of Sciences, Beijing 100049, China; 3 School of Material Science and Engineering, Shanghai University, Shanghai 200444, China; 4 College of Materials Science and Opto-Electronic Technology, University of Chinese Academy of Sciences, Beijing 100049, China

**Keywords:** polymer-matrix composites (PMCs), carbon fiber, carbon nanotubes, fracture toughness, fiber bridging

## Abstract

Carbon fiber reinforced polymer composites have the advantages of being lightweight, having high strength and designability, and having been extensively used. However, the interlaminar toughness and delamination resistance of these composites are relatively poor due to their laminated structure and intrinsic brittleness of resin matrix. In this paper, commercialized free-standing carbon nanotube (CNT) films, drawn from CNT forests, were used to toughen the interlaminar interfaces of the composites. The effects of resin infiltration state and thickness of CNT films on the interlaminar toughening effect were systematically investigated. The results show that the pre-infiltration treatment of CNT films with acetone diluted epoxy resin solution can effectively improve the degree of resin infiltration. Compared with the samples containing untreated CNT film, the Mode I and Mode II interlaminar fracture toughness of the treated samples were significantly improved. The G_IC_ reached a maximum of 1412.42 J/m^2^ at a CNT film thickness of 5 µm, which was about 61.38% higher than that of the baseline. At a CNT film thickness of 15 µm, the G_IIC_ reached a maximum value of 983.73 J/m^2^, approximately 67.58% higher than that of the baseline. The corresponding toughening mechanisms were also systematically analyzed.

## 1. Introduction

Carbon fiber reinforced polymer (CFRP) composites combine the advantages of both fiber and resin components and thus have properties, such as being stronger than steel and lighter than aluminum and having excellent designability. Thus, it can be popularly used in many fields, for instance, aerospace, military, and industry [1]. The superior mechanical properties of composite laminates along the axial direction derive from the in-plane arrangement of fibers [2]. However, instead of fibers in the interlayer along the out-of-plane direction, a more brittle resin is present and the resin matrix in a composite is usually the first to fail [3,4]. Furthermore, due to its high crosslink density, it would be less resistant to the initiation and growth of cracks [5], and the laminates have poor interlaminar toughness along the thickness direction. They are prone to delamination and cracking under loads, such as in-plane compression, bending, fatigue, and transverse impact, which greatly limit the application of fiber-reinforced composites. Therefore, improving the interlaminar fracture toughness and delamination resistance of CFRP is of great significance for the whole field of CFRP applications [6,7].

Many methods have been put forward to suppress delamination, such as 3D woven [8], Z-pinning [9], laminate stitching [10], and edge cap reinforcement [11]. Nevertheless, these methods can lead to increased manufacturing costs, increased mass, and loss of in-plane properties of the composites [12]. In recent years, an interlaminar toughening technique, interleaving, has been proposed, which is based on “ex-situ” toughening [13]. The toughened material is interleaved into the interlayer region of laminates, which can improve the fracture toughness of the laminate by promoting the formation of mechanical connections between the crack interfaces as well as the zigzag crack path [14,15]. 

The interleaving techniques studied so far can be mainly classified as particles [16,17], fibers [18], and films [3,19]. Among them, CNT films have a unique high specific surface area and outstanding flexibility. Many methods can be used for the preparation of CNT films, such as floating catalytic chemical vapor deposition (FCCVD) [20], Langmuir–Blodgett (LB) technique [21], electrophoretic deposition (EPD) [22], layer-by-layer (LBL) assembly [23], and so on. Additionally, it can be easily laid between the fiber layers before resin infusion or directly inserted into prepregs, which is compatible with the standard manufacturing process of FRP composites. Therefore, CNT films are considered to be excellent interleaf materials for interlaminar toughening application. In addition, CNT in CNT film can impede crack expansion by pulling out, fracturing, or bridging [24] and play the role of CNT matrix toughening, while avoiding the problems of resin viscosity increase and uneven dispersion of CNTs when directly mixing CNT powders with resin [25,26]. 

For example, Xu et al. [3] used the floating catalytic chemical vapor deposition (FCCVD) [27] method to deposit CNT film directly on carbon fiber fabrics for improving the interlaminar properties. The results showed that the bending strength and interlaminar shear strength of the composites increased by 16.04% and 21.51%, respectively, when the mass fraction of CNT films was 0.22%. Ou et al. [20] deposited low-density CNT films directly on woven carbon fabrics by the FCCVD method, and the Mode I interlaminar fracture toughness was improved by as much as 60%. Yu et al. [28] obtained a series of CNT films with different areal densities by controlling the deposition time. They found that the Mode II interlaminar fracture toughness of the composite laminates improved with the increase of CNT surface density in a certain range. In addition, the best Mode II interlaminar fracture toughness of the laminates was obtained when the surface density of CNT films was 9.64 g/m^2^, which increased by 94% compared with the standard sample. However, the interlaminar fracture toughness decreased when a certain critical value is exceeded. This is most likely related to the poor resin impregnation of CNT films [29]. Permeability is used to evaluate the level of difficulty of resin infiltration. In contrast to the fiber preforms that have a micro-scale pore structure, CNT films normally have a nanoscale pore structure, and their permeability is affected by pore shape, pore size, temperature, and curvature of the flow path, which is about 10^−19^–10^−17^ m^2^ [30,31,32,33,34]. For this reason, the resin with relatively low viscosity can easily penetrate the fiber network. However, it is inhibited in penetrating the CNT film. The resin macromolecules are blocked when flowing through the nanoscale pores of the CNT film, creating a blocking effect, that is to say the flow rate decreases during the penetration of the resin solution, resulting in the inability of the resin to completely infiltrate the CNT network within a limited gel time window of resin during infusion [34]. 

In our previous works, we have proposed two effective methods to solve this infiltration issue. One is using non-densified (as-received) fluffy CNT fiber veils [20], which can maintain a relative high permeability during resin infusion. Another is adopting ultra-thin CNT films (100–300 nm) [35], which can ensure full infiltration by resin for a limited period of time regardless of high or low permeability. However, both methods require the direct deposition of CNTs on the surface of carbon fabrics since their respective CNT assemblies are not free-standing, causing inconvenience in transportation and storage, thus affecting their further commercial application. Certainly, if CNT films are already tightly incorporated onto the carbon fabric or prepreg during the manufacturing process, the extra step of interleaving in composite layup is removed. In other words, carbon fabric/prepreg with CNT films can be used just as normal fabric/prepreg in the construction of composite structures. 

Reducing the viscosity of the permeating liquid is also a possible solution for the above-mentioned infiltration issue. Wang et al. [34] investigated the permeation behavior of different liquids in bucky papers. The results showed that when water and acetone were used as the permeating liquids, the permeation rates of bucky papers were similar within the error range. When acetone solution of epoxy resin was used as the permeating liquid, the permeation rate of bucky papers was lower than that of water and acetone tests but still higher than that of pure resin as the permeating liquid. This indicates that mixing the resin with a solvent with higher permeability can effectively reduce the viscosity of the resin solution, which helps the resin molecules pass through the pores and reduce “clogging” to improve the infiltration effect of the resin. For example, Li et al. [29] prepared nanocomposites of CNT films and epoxy resin by immersing the CNT films in a mixture of epoxy resin and acetone. The resin content in the composite film was influenced by controlling the mass ratio of epoxy resin to acetone, which was used to study the effect of resin content on the mechanical properties of nanocomposites. This behavior inspired us to make a breakthrough in the problem of infiltration of CNT films with large thickness. For this reason, after using 5 µm CNT film for interlayer toughening, it was found that its interlaminar toughening effect was not satisfactory due to insufficient infiltration. We chose acetone as a highly permeable liquid mixed with epoxy resin and treated CNT film by soaking in an epoxy resin/acetone solution before using it as a toughened interlayer for interlaminar toughening. 

In this paper, acetone was chosen as the high permeability solution and mixed well with epoxy resin. Then, three different thicknesses of CNT films (5 µm, 10 µm, 15 µm) were selected and soaked in epoxy resin/acetone solution. The laminated composites were prepared by vacuum-assisted resin transfer molding (VARTM). Double cantilever beam (DCB) and end-notch bending (ENF) tests were used to evaluate the interlaminar fracture toughness of the laminates. Then, the impact of resin infiltration state and thickness on the interlaminar toughening effect was determined by comparing them with the baseline specimens and untreated CNT film interlaminar-toughened specimens, respectively. To investigate the toughening mechanism of CNT films, the fractured cross-sections and fracture surfaces of the specimens were analyzed using ultra-deep field 3D microscopy and electron microscopy separately. 

## 2. Materials and Methods

### 2.1. Materials

Commercial non-crimped unidirectional carbon fabrics (12 k) with an areal density of 300 gsm were purchased from Toray Co., Ltd. (Hongkong, China). EPON™ Resin 862 (diglycidyl ether of bisphenol F) was acquired from Hexion, Inc. (Columbus, OH, USA). D-230 (polyetheramine hardener) was obtained from Maitu Chemical, Co., Ltd. (Weifang, China). CNT films were drawn from vertically aligned CNT forests, which were acquired from Shenzhen Core Technology Co., Ltd. (Shenzhen, China). In this study, 3 types of CNT films were selected with thicknesses of 5, 10, and 15 μm, respectively.

### 2.2. Preparation of CNT-Film-Toughened Interlayer

**Figure 1:**
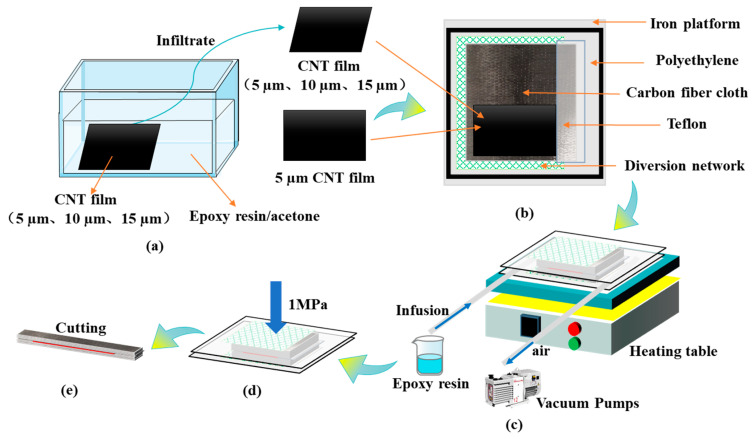
Flow chart of sample preparation. (**a**) Treatment of CNT films; (**b**) Laying of carbon fiber preforms; (**c**) Resin infusion; (**d**) Curing and molding; (**e**) Cutting plates.

The curing agent, D-230 (mass: 35.2 g), was first poured into a beaker containing the EPON 862 epoxy resin (mass: 100.0 g) and stirred well to mix them uniformly. Then, the acetone with the mass of 70.0 g was added and stirred well. After that, the CNT films (12 cm × 18 cm) of different thicknesses (5 µm, 10 µm, 15 µm) were soaked in epoxy resin/acetone solution for 30 min at room temperature, as shown in Figure 1a. After the excess epoxy resin on the surface of CNT films was removed, the acetone inside was evaporated by placing the films in a vacuum oven at 60 °C for 10 min.

### 2.3. Preparation of Laminates

Firstly, the unidirectional carbon fabric was cut into a 25 cm × 25 cm square and laid up sequentially to form a preform configuration of [0]_12_ in which a CNT film was interleaved in the middle as shown in Figure 1b. A Teflon film (for pre-cracking) was also inserted in the midplane to serve as an initiation site of the delamination. The specimens containing 5 µm dry CNT film are denoted as CNTF_5D_. The baseline and the specimens containing pre-wetted CNT films with thicknesses of 5, 10, and 15 µm are denoted as CNTF_0_, CNTF_5_, CNTF_10_, and CNTF_15_, respectively.

In this experiment, the epoxy resin (mass: 270.4 g) was infused into the preform using the vacuum-assisted resin transfer molding (VARTM) process, as shown in Figure 1c, and the resin used for infusion was the same as that used in the pre-wetting treatment of CNT films. After the infusion was completed, the preforms were transferred to a flat vulcanizer and cured at 80 °C/1 MPa for 2 h and 120 °C/1 MPa for 2 h with a warming process of 20 min.

### 2.4. Characterizations

#### 2.4.1. Interlaminate Fracture Toughness Tests

The Mode I and Mode II interlaminar fracture toughness were tested on the AI-7000-LAU10 universal material testing machine, respectively. The Mode I interlaminate fracture toughness of the composite laminate was tested by double cantilever beam test (DCB) at room temperature according to ASTM D5528-01 [36] with a sample size of 240 mm × 21 mm × 3.5 mm. In the first stage of the test, the specimen was pre-loaded to obtain 50 mm pre-cracks to minimize the influence of the non-adhesive insert on the measured fracture toughness [35]. The specimens were reloaded at a rate of 1 mm/min in the second phase of the test to extend the crack from 50 mm to 100 mm and then unloaded. The Mode I interlaminar fracture toughness was calculated as follows:

$$G_{IC}=\frac{3P\delta }{2b(a+\left|∆\right|)}$$

where G_IC_ is the Mode I interlaminar fracture toughness; the P is the load; δ stands for the load point displacement; b represents the specimen width; and a is the delamination length. The ∆ can be determined experimentally by generating a least squares plot of the cube root of compliance, C^1/3^, as a function of delamination length.

The Mode II interlaminar fracture toughness of composite laminates was tested by the end-notched flexure (ENF) test according to ASTM D7905 [37] at room temperature with a sample size of 240 mm × 21 mm × 3.5 mm and a loading rate of 0.5 mm/min. The Mode II interlaminar toughness was calculated as follows:

$$G_{IIC}=\frac{3mP_{max}^{2}a_{0}^{2}}{2B}$$

where G_IIC_ is the Mode II interlaminar fracture toughness; m is the CC coefficient; $P_{max}$ is the maximum value of force on the load-displacement curve; a_0_ is the crack length; and B is the specimen width.

#### 2.4.2. Other Characterizations

A scanning electron microscope (Hitachi Regulus 8230, Tokyo, Japan) was used to observe the morphology of the fracture surfaces of the composite laminate after the DCB test and the accelerating voltage of the SEM under which the imaging is 4.0 kV. Super depth of field 3D microscope (VHX-7000, Keyence, Osaka, Japan) was utilized to observe the cross-section of the laminate.

## 3. Results and Discussion

### 3.1. Mode I Fracture Test

**Figure 2:**
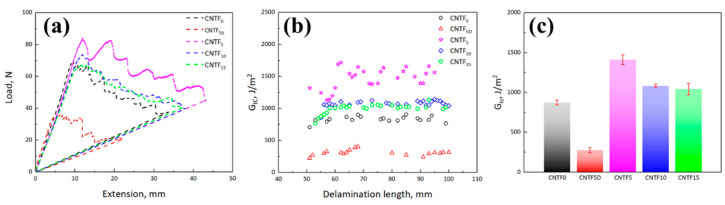
(**a**) Load-extension curves; (**b**) R-curves; (**c**) comparison of Mode I interlaminar fracture toughness of different specimens: CNTF_0_, CNTF_5D_, CNTF_5_, CNTF_10_, and CNTF_15_.

Figure 2 exhibits the representative data from the Mode I fracture tests. It can be found that the G_IC_ values of the samples containing treated CNT film are higher than that of CNTF_0_ (Figure 2c), indicating that they could delay the crack expansion, improving the critical load. In contrast, the critical load and G_IC_ value of the samples containing untreated CNT film (CNTF_5D_) are significantly lower than that of CNTF_0_ (Figure 2a,c). This indicates that the untreated CNT film cannot achieve the effect of interlaminar toughening. Among them, CNTF_5_ has the highest G_IC_ and the R curve (Figure 2b), exhibiting distinct fluctuations compared to other samples. 

Noting that whether CNT film is treated with the epoxy resin/acetone solution by soaking or not has a great influence on its toughening effect. The resin molecules are large sizes, and the CNT film has nanoscale pores. Therefore, when the resin reaches the CNT film zone, it will be difficult to penetrate and easy to block and enrich around it. If the connection in the CNT film layer is not firm, it is not conducive to improving its interlaminar fracture toughness. This is also the reason why CNTF_5D_ cannot achieve the toughening effect. After adding acetone to the epoxy resin, the viscosity of the resin solution is greatly reduced [34]. The resin macromolecules in the epoxy resin/acetone solution can enter the pores of CNT film along with acetone without “blocking”, which effectively infiltrates the CNT film and reduces the resin enrichment zone. The results demonstrated that the G_IC_ value of the treated specimen CNTF_5_ was more than 5.1 times higher than that of CNTF_5D_ and more than 1.6 times higher than that of CNTF_0_, increasing considerably in the interlaminar fracture toughness. The specific values of G_IC_ are shown in Table 1.

**Figure 3:**
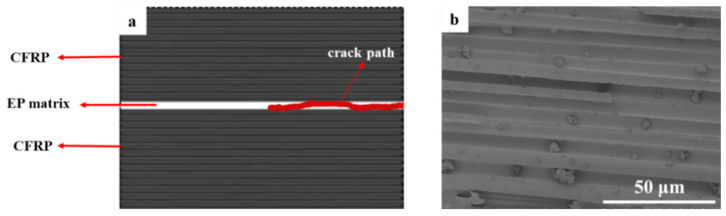
(**a**) Schematic diagram of fracture mechanism and (**b**) SEM image of fracture surface of CNTF_0_.

Toughening mechanisms can be revealed by observing their fractured surfaces and interfaces. As shown in Figure 3b, many complete resin pits are left by the debonding of carbon fibers on the CNTF_0_ crack surface. Furthermore, the delamination surface is very clean and smooth with limited fiber bridging, indicating that the crack expansion is in a relatively stable path and always being confined to the interlaminar region (see Figure 3a), which corresponds to its flat R-curve and low G_IC_ (875.23 J/m^2^). It can be attributed to the interlaminar structure of CNTF_0_, which only relies on the resin matrix to bond to each other. As a result, it would cause the debonding failure under the Mode I loading condition [38]. 

**Figure 4:**
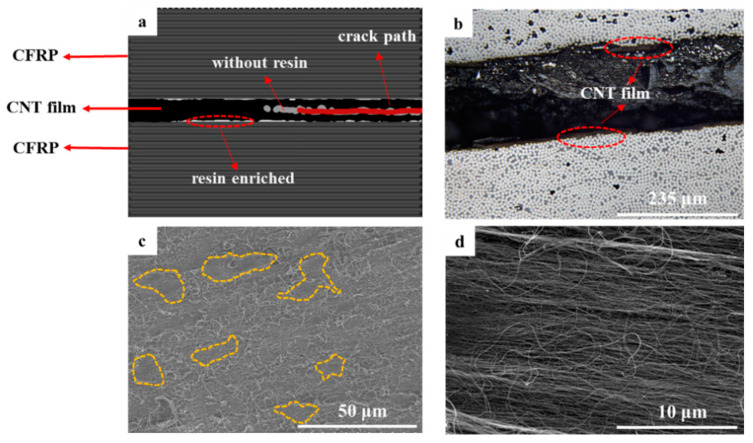
(**a**) Schematic diagram of fracture mechanism; (**b**) optical microscopy image of fractured section; (**c**) SEM image of fracture surface of CNTF_5D_; (**d**) SEM image of 5 µm CNT dry film.

The SEM image of the 5 µm CNT film is shown in Figure 4d. The CNT film is very dense, and the gap between the fibers is extremely narrow. Figure 4c displays the fracture surface of the CNTF_5D_ laminate with most of the CNTs exposed, indicating that an unpenetrated region would be formed in the CNT film and cracks will spread at the interface. In addition, only a small amount of resin enrichment zone can be distinguished (yellow dashed line), suggesting the poor infiltration of the resin into the dense CNT film. The cracks would easily expand in this region with slight energy consumption. As shown in Figure 4b, CNTs are present at both ends of the crack. This indicates that the crack is “confined” to this CNT film and hardly cross-connects with the carbon fiber–epoxy interface (see Figure 4a), which in turn severely limits fiber bridging. Therefore, CNT film in CNTF_5D_ cannot play the role of interlaminar toughening, and it will act as a defective layer to make cracks expand in it easily, causing the decrease of interlaminar fracture toughness.

**Figure 5:**
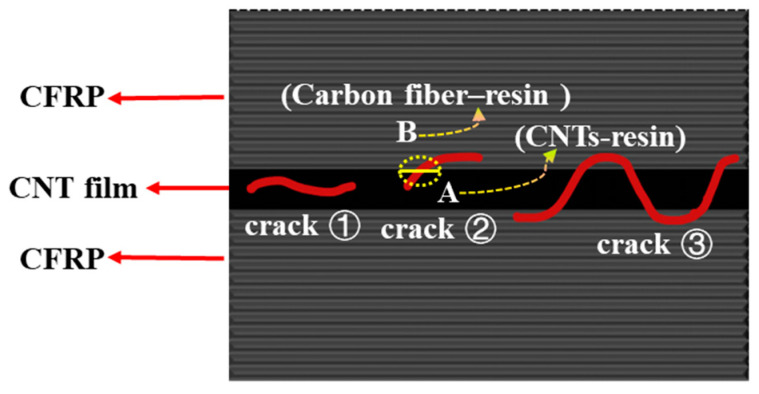
Schematic diagram of fracture mechanism in the samples containing pre-wetted CNT film.

Different from CNTF_5D_, the addition of acetone enables all the CNT films in CNTF_5_, CNTF_10_, and CNTF_15_ to be well infiltrated. As a result, the interlaminar fracture toughness of the samples containing the treated CNT films was significantly improved, with CNTF_5_ having the best toughening effect. Regarding its fracture mechanism, it can be summarized as follows (as shown in Figure 5): crack ① expands completely within the interlaminar zone; crack ② deflects from the interlaminar zone to the intra-laminar zone; and crack ③ penetrated through the whole CNT-film-toughened layer, forming “interlaminar crossing”. 

**Figure 6:**
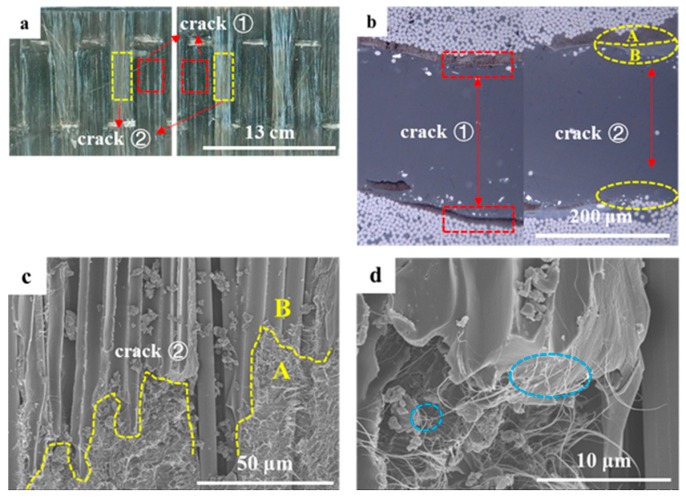
(**a**) Image of fracture surface of CNTF_5_; (**b**) optical microscopy image of fractured cross-section of CNTF_15_; (**c**,**d**) SEM image of fracture surfaces of CNTF_5_.

“Interlaminar propagation” is a typical fracture mechanism observed in CNTF_5_, CNTF_10_, and CNTF_15_ (crack ①). It can be observed that the presence of CNTs is in both fracture surfaces of CNTF_5_ (Figure 6a, red dashed boxes) as well as at both sides of crack in the CNTF_15_ (Figure 6b, red dashed boxes), which indicates that the crack propagates within the CNT film layer. Compared to CNTF_0_, the cracks would expand more tortuously and consume more energy in the inner region of the CNT film containing the resin. In addition, CNTs will also play a role similar to that of matrix toughening, where CNTs are pulled out from the resin matrix or fracture (see Figure 6d, blue dashed boxes). Cracks ② (yellow dashed box in Figure 6b) are mainly observed in CNTF_10_ and CNTF_15_. It displays the intact CNT film on one side of the crack. Figure 6c shows two regions, which are A (CNTs-resin region) and B (carbon fiber–resin region), with an obvious demarcation line between them (yellow dashed line). This indicates that the crack will be deflected from the CNT-toughened region to the “baseline-like” intra-laminar region, and interfacial debonding would occur. As the thickness of CNT film increases to a certain extent, the toughened layer in the interlaminar region is continuous, thick, and hard. This indicates that cracks generated in this region have a hard time penetrating through the CNT-film-toughened layer. Once the crack is deflected from the tougher interlaminar-toughened region of the CNT film to the relatively weaker intra-laminar region [35], it continues to expand at the interface between the resin matrix and the carbon fiber.

**Figure 7:**
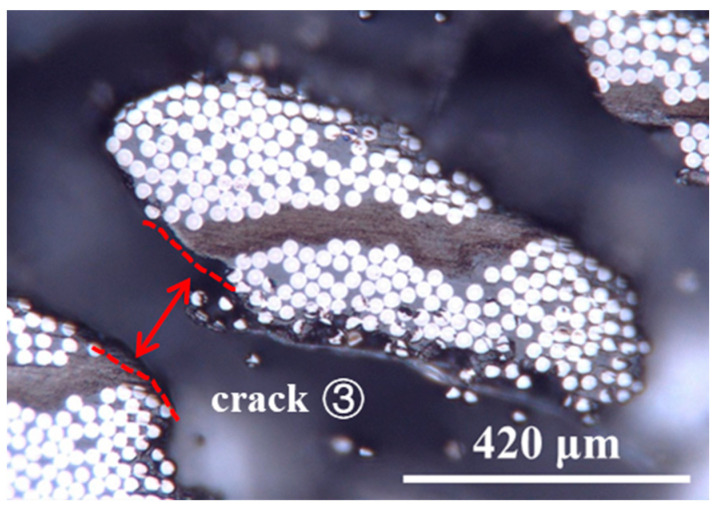
Optical microscopy image of fracture section of CNTF_5_.

CNTF_5_ has a different crack propagation mode from CNTF_10_ and CNTF_15_, as shown in Figure 5, Crack ③. Figure 7 displays the optical microscopy image of fracture section of CNTF_5_. It can be seen that the cracks departed from the carbon fiber–resin layer on one side to the other side, crossing through the entire CNT film layer. In addition, the alternation of carbon fiber–resin region and CNTs–resin region (see Figure 6a) suggests that the cracks can deflect back to the CNT film interlaminar-toughened region after deviating to the intralaminar region (see Figure 5, crack ③). It is very favorable for the initiation of fiber bridging. In this case, the failure mechanism of CNTF_5_ is not only interlaminar crossing but also micro-scale fiber bridging and nano-scale fracture and pullout of the CNT. This would form a multiscale toughening with a better synergistic effect; thus, its Mode I toughening is the best.

### 3.2. Mode II Fracture Test

**Figure 8:**
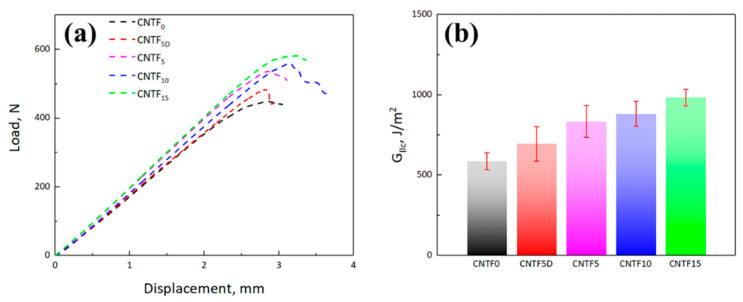
(**a**) Load-defection curves; (**b**) comparison of Mode II interlaminar fracture toughness of different specimens: CNTF_0_, CNTF_5D_, CNTF_5_, CNTF_10_, and CNTF_15_.

Figure 8a displays the typical load-displacement curves for the Mode II fracture tests. It can be observed that all samples possess typical brittle behavior under Mode II loading. At the initial stage, the load increases linearly with increasing displacement until the maximum was produced. Then, the load dropped suddenly because of the initiation of the crack. In the Mode II fracture test, the laminate is subjected to interlaminar shear forces that cause delamination by inconsistent bending in the thickness direction, and cracks extend in the direction of the fibers [39]. Thereby, unlike the results of the Mode I fracture test, it can be found that the insertion of the CNT-film-toughened interlayer could improve the maximum loads for both untreated and treated CNT film-interleaved laminates, indicating all of them have the great toughening effect.

Figure 8b compares the average G_IIC_ value of the different samples. The mean G_IIC_ for CNTF_0_ is 587.00 J/m^2^, while the CNTF_15_ shows the most significant toughening effect with an improvement of about 67.59% compared to CNTF_0_. Unlike the Mode I case, the G_IIC_ value of CNTF_5D_ was still higher than the baseline, which should be ascribed to the different stress states with Mode I being dominated by peel stress and Mode II being prevailed by shear stress, knowing that dry CNT film can withstand high in-plane friction but less able to resist out-of-plane peeling. In addition, the G_IIC_ value increased with the increasing the thickness of CNT film, showing a totally different trend from G_IC_. The specific values of G_IIC_ are shown in Table 2.

**Figure 9:**
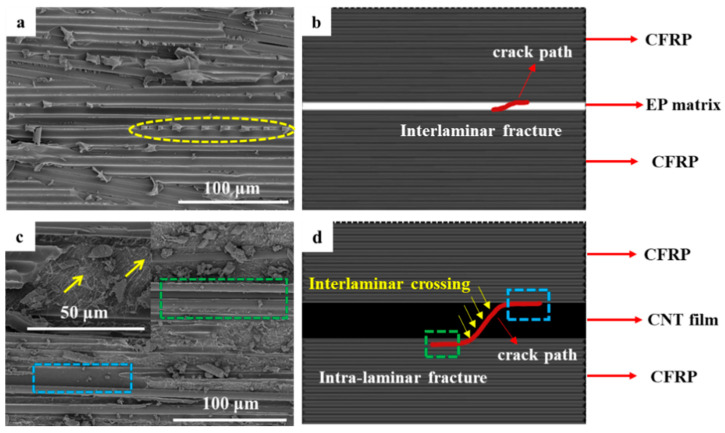
(**a**) SEM images of fracture surface of CNTF_0_; (**b**) schematic diagrams of fracture mechanism of baseline; (**c**) SEM images of fracture surface of CNTF_5_; (**d**) schematic diagrams of fracture mechanism of the specimens containing CNT film.

As shown in Figure 9a, pre-cracking of CNTF_0_ without a toughened interlayer may exist at the carbon fiber–resin interface. When the crack starts to expand, interfacial debonding would occur at the carbon fiber–resin interface, and the carbon fiber can be pulled out from the resin leaving a crater. It can be observed that shear fracture would occur under Mode II loading in the pure resin matrix between the carbon fibers, as shown by the yellow dashed box in Figure 9a, which exhibits a characteristic shear pattern. Therefore, the fracture modes of the baseline are mainly interfacial debonding and matrix shear, which belong to interlaminar fracture [40] (Figure 9b). 

Under Mode II loading, interfacial debonding would occur at the interface between the carbon fiber–resin and the toughened layer (Figure 9c, blue dashed box) in the specimens containing the CNT film. It can be found that the fracture surface is relatively smooth, and the presence of CNT indicates that the crack can expand along the interface. In addition, debonding at the interface between the carbon fibers and the resin can also be observed (Figure 9c, green dashed box), indicating that the cracks would deflect outward into the intra-laminar region. The cracks may cross through the entire toughened layer of the CNT film (Figure 9c, yellow arrows), resulting in “interlaminar crossing”. The fracture mechanism diagrams for these two interfacial debonding methods are shown in Figure 9d, blue and green dashed box. Unlike the pure resin matrix shear of the baseline, CNT films fully infiltrated by the resin can consume a lot of energy during crack expansion through the pullout and fracture of CNTs in the resin matrix [28]. In addition, crack expansion tends to split the CNT film into irregular shapes, and the expansion path will be more tortuous (Figure 9d), thereby consuming more energy. In short, besides interfacial debonding, the fracture mode of the specimen containing CNT films also includes interlaminar crossing with abundant micro-scale CNT bridging as well as intra-laminar fracture with substantial macro-scale carbon fiber bridging, leading to a remarkable enhancement of fracture energy.

## 4. Conclusions

This paper proposed a facile and scalable method to toughen the interlaminar region of unidirectional CFRP laminates. A comparative study was performed to systematically explore the effects of resin infiltration and interleaf thickness on the interlaminar-toughening efficiency of CNT films. The following conclusion remarks can be drawn:

The G_IC_ value of CFRP is degraded a lot when a 5-µm-thick dry CNT film is interleaved, which should be ascribed to the poor resin impregnation of CNT films. After soaking the CNT film with the epoxy resin/acetone solution, the infiltration of resin can be effectively improved, thus significantly enhancing the interlaminar fracture toughness of resultant laminates. The results showed that the G_IC_ value of the treated specimen CNTF_5_ was about 5.1 times higher than that of CNTF_5D_ and 1.6 times higher than that of baseline. 

For the three different thicknesses of CNT film, the toughening effect of CNTF_5_ was the most obvious under the Mode I loading condition, with an improvement of about 61.38% compared with the control. The Mode I toughening effect of CNT films gradually decreased with increasing thickness within the range of 5–15 µm, but they were all higher than the baseline. In comparison, the Mode II toughening effect of CNT films increased with increasing the film thickness, and the highest improvement (67.58%) was achieved when 15-µm-thick CNT film was interleaved. The difference in the toughening efficiencies of CNT film for Mode I and Mode II fracture at different thicknesses should be attributed to their respective distinct toughening mechanisms.

## Figures and Tables

**Table 1:** Summary of Mode I interlaminar fracture toughness test results.

Sample	CNTF_0_	CNTF_5D_	CNTF_5_	CNTF_10_	CNTF_15_
G_IC_ (J/m^2^)	875.23 ± 31.75	277.74 ± 31.96	1412.42 ± 60.99	1083.25 ± 19.47	1042.93 ± 73.09
Improvement	—	−68.27%	+61.38%	+23.77%	+19.16%

**Table 2:** Summary of Mode II interlaminar fracture toughness test results.

Sample	CNTF_0_	CNTF_5D_	CNTF_5_	CNTF_10_	CNTF_15_
G_IIC_ (J/m^2^)	587.00 ± 54.13	694.41 ± 106.56	835.05 ± 100.22	882.62 ± 78.59	983.73 ± 51.04
Improvement	—	+18.30%	+42.26%	+50.36%	+67.59%

## Data Availability

The data that support the findings of this study are available from the corresponding authors [Y.O. and D.M.], upon reasonable request.
